# A Rare Case of Cutaneous Metastasis of Bladder Transitional Cell Carcinoma

**DOI:** 10.5826/dpc.1104a108

**Published:** 2021-10-01

**Authors:** Tahel Fachler, Diana Prus, Amichay Meirovitz, Yuval Ramot

**Affiliations:** 1 Department of Dermatology, Hadassah Medical Center, Hebrew University of Jerusalem, The Faculty of Medicine, Jerusalem, Israel; 2 Department of Pathology, Hadassah Medical Center, Hebrew University of Jerusalem, The Faculty of Medicine, Jerusalem, Israel; 3 Department of Oncology, Hadassah Medical Center, Hebrew University of Jerusalem, The Faculty of Medicine, Jerusalem, Israel

**Keywords:** dermato-oncology, cutaneous metastasis, transitional cell carcinoma, bladder cancer

## Introduction

Cutaneous metastases originating from a primary solid visceral organ are an uncommon phenomenon, seen in 0.3% to 9% of patients [1]. Cutaneous metastases from a urinary origin are especially rare, reported in only 1.1% of urologic malignancies [1]. Although urothelial carcinoma is the third most prevalent malignancy in adults [2], cutaneous metastases from a genitourinary origin account for only 10.4% of cutaneous metastases. The current report provides a detailed description of a case with this rare condition.

## Case Presentation

An 81-year-old man presented with a painful and tender lesion on his right arm, that had been growing for the last month. He had a clinical history of metastatic bladder transitional cell carcinoma (TCC), for the past 7 years, which was treated with radical cystectomy, chemotherapy, and immunotherapy. Two years prior to admission he was treated with irradiation to the right humerus due to bone metastasis. At the time of presentation his disease was partially controlled with immunotherapy (pembrolizumab). He suffered from chronic lymphedema of the right arm due to radiotherapy-induced lymphatic injury, and he underwent a thrombectomy of an arterial occlusion on the same arm 1 month prior to presentation. He had no previous dermatological pathologies.

**Figure 1:**
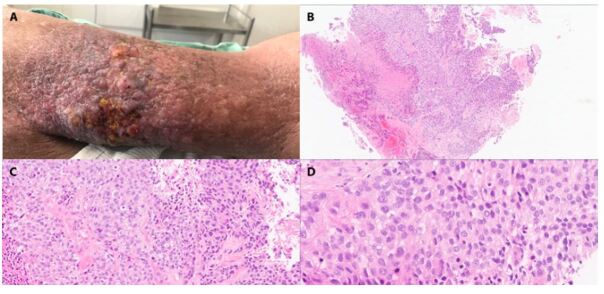
(A) An indurated and ulcerated plaque on the right arm of an 81-year-old man. (B–D) Histopathology images of a skin biopsy taken from the lesion, showing major dermal infiltration by poorly differentiated carcinoma cells with focal squamous differentiation and extensive necrosis (H&E, (B) magnification X5, (C) magnification X20, (D) magnification X40).

**Figure 2:**
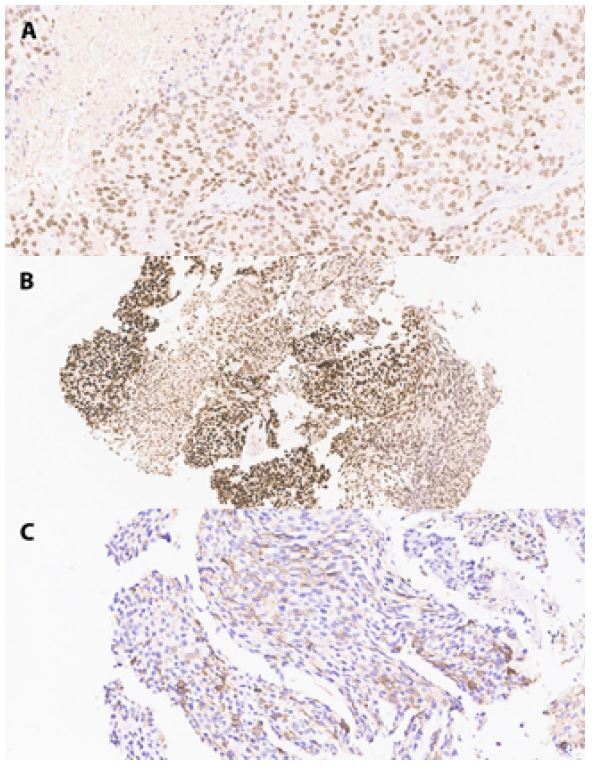
Immunohistochemistry of the skin biopsy taken from the right arm. (A) GATA-3. (B) p40. (C) CK20 (X200).

Clinical examination revealed a hardened plaque on the extensor aspect of the right arm, composed of coalescing erythematous-to-purple nodules, with partial ulceration in the middle of the lesion (Figure 1A). The surrounding skin was edematous and swollen, with pigmentary changes consistent with radiotherapy-induced damage. A skin biopsy (Figures 1, B–D and 2) revealed subcutaneous tissue infiltrated by poorly differentiated carcinoma cells with focal squamous differentiation and extensive necrosis (Figure 1 B–D). Immunohistochemical analysis showed GATA-3, p40 and CK20 positive staining (Figure 2). Based on the histological and immuno-histochemical findings, the diagnosis of metastatic urothelial carcinoma was made.

## Conclusions

Cutaneous metastases have been reported more commonly in middle-aged and older men compared to women, and in most patients, they convey a grave prognosis, with patients typically surviving only a few weeks [2]. Metastatic dissemination can occur by direct invasion, implantation on operative scars, and lymphatic or hematogenous spread [1]. In our patient, the latter is the most likely mode of dissemination due to focal vascular invasion that was observed in the primary tumor.

Cutaneous metastases in urothelial carcinoma pose a diagnostic challenge due to their infrequent occurrence, in addition to being further complicated by the long development period, usually several years after curative therapy [2]. Additionally, they can have variable non-specific clinical presentations, such as urticarial, “nonspecific” macular rash [1], large erythematous indurated plaques, and other manifestations. Differential diagnoses include radiation dermatitis, lymphedema, and lymphangiectasis, in addition to cutaneous drug reaction and opportunistic infections.

In this report, we presented a rare case of cutaneous metastasis from TCC, at a progressive stage of a known metastatic malignancy. In this case, progressive disease was already present prior to the diagnosis of the dermal lesion. However, in 23.3% of cutaneous metastases, skin metastases are the first indication of malignancy. This report emphasizes the need for a cautious approach when assessing these lesions, in order to rule out metastasis at the different phases of malignant disease.
